# The use of *Nibima* and *Immunim*, two Ghanaian herbal medicines in the management of mild COVID‐19: A case report

**DOI:** 10.1002/ccr3.8539

**Published:** 2024-02-23

**Authors:** Mavis Boakye‐Yiadom, Ronald Yeboah, David Offei‐Abrokwa, Ekuwa Baisiwa Wilson, Samuel Annang Darko, Emmanuel Tamakloe

**Affiliations:** ^1^ Clinical Research Department Centre for Plant Medicine Research Mampong‐Akuapem Ghana

**Keywords:** COVID‐19, herbal medicine, *Immunim*, *Nibima*, SARS‐CoV‐2

## Abstract

**Key Clinical Message:**

*Nibima* and *Immunim* manages mild COVID‐19 in 7 days.

**Abstract:**

Coronavirus Disease 2019 (COVID‐19), caused by Severe Acute Respiratory Syndrome Coronavirus 2 (SARS‐CoV‐2) can lead to severe complications and deaths. The search for phytotherapeutic agents to augment the fight against the current COVID‐19 pandemic is therefore of highest priority. A 52‐year‐old female with no history of chronic illness presented to our clinic facility with a 3 days loss of smell and headache which persisted after self‐medication. The patient tested positive for COVID‐19 in the SARS‐CoV‐2 Ag test as well as the Typhoid rapid antibody test. Routine laboratory tests were not remarkable. A 60 mL three times daily dose of *Nibima* and 5 mL in 40 mL of warm water dose of *Immunim* were given to the patient for 7 days. Patient recovered sense of smell and regained appetite but cough although reduced persisted. She also tested negative for COVID‐19 after 7 days treatment. This case is the first documented case of COVID‐19 management with herbal medicines in Ghana. We strongly suggest a larger control trial on these products to ascertain these findings to repurpose them as viable treatments for COVID‐19.

## 1 BACKGROUND

The emergence of the recent novel coronavirus (COVID‐19) has seen a rapid increase in infected persons across the globe. The disease was declared a global pandemic by WHO in March, 2020 upon its rapid transmission rate.^1^ COVID‐19 is a newly identified Severe Acute Respiratory Infection (SARI) caused by the novel coronavirus named Severe Acute Respiratory Syndrome Coronavirus 2 (SARS‐CoV‐2) which belongs to the Coronaviridae family. These viruses are enveloped viruses with a positive‐sense single‐stranded RNA genome in nature. Most human coronavirus infections are mild, the epidemic beta‐coronaviruses, severe acute respiratory syndrome coronavirus (SARS‐CoV) and Middle East respiratory syndrome coronavirus (MERS‐CoV), caused over 10,000 cases in two decades with 10% and 37% mortality rates for SARS‐CoV and MERS‐CoV respectively.^2^, ^3^ SARS‐CoV‐2 is able to infect host by binding between the viral structural spike proteins and the angiotensin‐converting enzyme 2 (ACE2) receptor, found in the lung, heart, intestinal, epithelium, vascular endothelium, and kidneys which result in a cascade of severity ranging from asymptomatic form to critical respiratory syndrome and heart failure which requires mechanical ventilation. Among symptomatic patients, about 80% exhibit mild symptoms like dry cough, sore‐throat, low‐grade fever, and malaise.^2^, ^4^

In Ghana, to diagnose Covid‐19, a nasopharyngeal and/or oropharyngeal swab for Real Time Polymerase Chain Reaction (RT‐PCR) is needed. Other supportive laboratory investigations such as full blood count, chest X‐ray, D‐dimer, liver function test, C‐reactive protein (CRP), and renal function test could be conducted. A standard treatment available for mild to critical presentation in all age populations is a combination of the following treatments: Hydroxychloroquine or Chloroquine, Azithromycin or Doxycycline, Methylprednisolone, Convalescent plasma, Tocilizumab, and Remdesivir.^4^ In recent years, vilobelimab (used to treat hospitalized COVID‐19 adults administered within 48 h of mechanical ventilation or extracorporeal membrane oxygenation), Molnupiravir (Lagevrio) and Nirmatrelvir with Ritonavir (Paxlovid) (used for immunocompromised patients) have been added.^5^ While data are available for adult patients with COVID‐19 and even to some extent pediatric patients, evidence‐based reports on the use of herbal medicines in its treatment remain very limited.

Following the COVID‐19 pandemic, the surge in use of phytomedicines though promising, brought to bear the real challenges of providing safe, effective, affordable, and accessible medicines to the populations. The weak health systems in many countries worldwide and in particular, Africa were exposed.^6^, ^7^ WHO therefore recommends the use of medicinal plants which have their safety profile and efficacy assessed before they can be considered as treatments for management of diseases.^7^ Potential candidates such as *Azadirachta indica (A. indica)* and *Cryptolepis sanguinolenta (C. sanguinolenta)* are being investigated as treatment for the management of COVID‐19 hence must be subjected to same safety and efficacy principles.

*Nibima* and *Immunim* are two Ghanaian herbal formulations produced under good manufacturing practices by the Centre for Plant Medicine Research (CPMR) using a classified formula. Other properties of these herbal medicines are as shown in Table 1.

**TABLE 1:** Properties of *Nibima* and *Immunim*.

	*Nibima*	*Immunim*
Description	A 330 mL amber bottled decoction (aqueous extract) of the dried roots of *Cryptolepis sanguinolenta* used for the management of Malaria	A 120 mL amber bottled tincture (hyroethanolic extract) of the dried leaves of *Azadirachta indica* used as an immune system support supplement
Food and Drugs Authority of Ghana Registration	FDA/HD1.20‐02086	FDA/HD.20‐11485

The antiviral, anti‐inflammatory, and antioxidant potentials of both plants are well established hence their choice as suitable candidates for the management of COVID‐19 is justified.^8^, ^9^, ^10^, ^11^

Here, we report a case of hyposmia, cough, and anorexia that are classic symptoms of COVID‐19 in an elderly female who tested positive for COVID‐19.

## 2 CASE PRESENTATION

A 52‐year‐old Ghanaian woman visited the out‐patient department of the Dr. Oku Ampofo Memorial Clinic of the CPMR in the Eastern region of Ghana in October, 2021. She presented with loss of smell and headache of 3 days duration. On direct questioning she admitted to having non‐productive cough, loss of taste, borborygmus, loss of appetite and denied having chest pain, orthopnoea, dyspnoea, rhinorrhoea, and insomnia. Her body temperature was 36.1°C and blood pressure was 130/80 mmHg. On examination there was no pallor nor jaundice and hydration was fair. Her chest examination was unremarkable. Further inquiries revealed she was not vaccinated against the COVID‐19. A provisional diagnosis of malaria and COVID‐19 was made. A written consent was taken from her.

A request of typhoid rapid antibody test, blood film for malaria parasites and a SARS‐CoV‐2 Ag test was made. Venous blood and nasopharyngeal swab were taken and samples prepared for testing.

The result of the laboratory investigations are as follows:

The antigen test for SARS‐CoV‐2 was positive, typhoid rapid antibody test reactive for both IgM and IgG, and blood film for malaria parasites was negative. She was prescribed 60 mL three times daily of *Nibima* and 5 mL in 40 mL of warm water daily of *Immunim* to treat the COVID‐19. She was counseled to practice self‐isolation and observe all other COVID‐19 safety protocols and report for retesting in a week. Her immediate family and close contacts were brought in for COVID‐19 testing.

Of the three who were present for testing; her 9‐ and 12‐year‐old daughters and husband, one (husband) tested positive for COVID‐19. He was a 55‐year‐old man who presented with nasal congestion and occasional cough. When asked about his vaccination status for COVID‐19, he disclosed that he was not vaccinated. Since other tests were negative, he was also placed on the same treatment regimen and asked to report back in a week.

On review, the SARS‐CoV‐2 Ag test was repeated for both patients and the results were negative. Her cough had subsided and only mild abdominal pains were present on review. The husband however had all symptoms completely resolved.

## 3 DISCUSSION

Three years after the first recorded cases of COVID‐19, the disease continues spreading rapidly worldwide. The introduction of various interventions such as vaccination, wearing of face masks, social distancing, frequent hand washing and testing, though effective have still not fully controlled the disease. With the emergence of new variants and their subsequent detection across the globe‐ (the more contagious Delta and now Omicron variants), it has become imperative that we also control the disease with efficient therapeutics to prevent life threatening complications and deaths.^12^

This is the first case report on the successful management of two patients with mild COVID‐19 using herbal preparations in Ghana after diagnosing with the SARS‐CoV‐2 Ag test. We acknowledge, however, that further sensitive and reliable testing should be done to confirm the presence of SARS‐CoV‐2 as well as the variant in the nasopharyngeal swab taken. This was not possible as the needed conditions for the sample to be kept and transported to the testing site was not available and cost implications for conducting a RT‐PCR made it impossible at that time.

The clinical manifestation of COVID‐19 varies from no symptoms to critical illness. An individual is said to have asymptomatic infection when there is a positive test result to a virologic test with no symptoms that is consistent with COVID‐19. Mild COVID‐19 is defined as an individual having various signs and symptoms of COVID‐19 such as fever, cough, sore throat, malaise, headache, muscle pain, nausea, vomiting, diarrhea, loss of taste, and smell but do not have shortness of breath, dyspnoea or abnormal chest imaging. Individuals classified as having moderate COVID‐19 show evidence of lower respiratory disease during clinical assessment or imaging and have an oxygen saturation (SpO_2_) ≥94% on room air at sea level. Severe COVID‐19 is diagnosed when one has SpO_2_ < 94% on room air at sea level, a ratio of arterial pressure of oxygen to fraction of inspired oxygen (PaO2/FiO2) < 300 mmHg, respiratory frequency > 30 breaths/min, or lung infiltrates >50%. Critical COVID‐19 is characterized by individuals having respiratory failure, septic shock, and/or multiple organ dysfunction.^13^

It has been reported that most COVID‐19 patients develop mild or uncomplicated illness: approximately 14% develop severe disease and require hospitalization and or oxygen support, and 5% of them require intensive care attention which poses a significant threat to human health.^3^, ^4^ Now the understanding of the virus and the distribution in the elderly and younger population is a bit unpredictable. Some adults are asymptomatic and others have <3% of the known presenting symptoms such as loss of taste or smell, cough, shortness of breath or difficulty breathing, sore throat, therefore making it difficult to diagnose. Non‐specific symptoms such as abdominal discomfort, pains and flatulence in infected people may also lead to undetected individuals in the populace.^4^, ^12^

The risk factor for the patients was their age, with no other underlying factors. Since their presenting symptoms were mild,^3^, ^13^ it was possible to initiate this herbal treatment to aid speedy recovery.

The choice of *Nibima* and *Immunim* as a regimen for treating these patients for COVID‐19 was based on their documented antiviral, anti‐inflammatory, antioxidant, antiplasmodial, and immunomodulating properties.^8^, ^9^, ^10^

Some on‐going clinical trials and other research on Cryptolepine, the main alkaloid in the product *Nibima* have exhibited high binding affinity with potential inhibitory activity towards two of the major prions of SARS‐COV‐2, the main protease and the RNA‐dependent RNA polymerase which makes it a viable candidate for the management of COVID‐19. Again, the main strategy for identifying new COVID‐19 drug candidates has been via drug repurposing, aided by high throughput screening of existing antiviral agents. This is true of chloroquine and quinine, two treatments for malaria. Therefore, the choice to repurpose *A. indica* and *C. sanguinolenta* which both contain alkaloids and used in the management of malaria is duly justified .^14^, ^15^

In a computational prediction study of SARS‐CoV‐2 structural protein inhibitors from both *A. indica* and *C. sanguinolenta*, their potential to inhibit the functionality of the envelope proteins and membranes was established.^14^

Also, Azadirachtin and Nimbolide are two free radical scavenging compounds responsible for the antioxidant properties of the plant *A. indica*. The anti‐inflammatory properties of *A. indica* are known to act through the regulation of proinflammatory enzyme activity including cyclooxygenase (COX) and lipoxygenase (LOX) enzymes.^16^ Other beneficial pharmacological activities of *A. indica* include antipyretic, immunomodulatory, and antimicrobial.

## 4 CONCLUSIONS

This is the first report of management of COVID‐19 with a plant‐based medicine in Ghana after detection of COVID‐19 with SARS‐CoV‐2 Ag test. The COVID‐19 may present with common diseases such as typhoid fever or malaria not just the typical pneumonia. This viral infection may go undetected due to lack of requisite testing kit and equipment like the RT‐PCR; therefore, clinicians should be aware of the non‐specific symptoms of COVID‐19 in the country. We recognize, however, the limitations of our means of exploration which would confirm fully the variant in this case report.

It is recommended that measures should be put in place to confirm and manage properly the diseases we treat at our facilities. We suggest that studies should be conducted to allow possible clinical trial of both herbal products used in the management of COVID‐19 in Ghana. Furthermore, none of the medical staff involved was subsequently found to be infected by SARS‐CoV‐2.

## AUTHOR CONTRIBUTIONS

**Mavis Boakye‐Yiadom:** Conceptualization; methodology; writing – original draft. **Ronald Yeboah:** Conceptualization; methodology; writing – original draft. **David Offei‐Abrokwa:** Writing – original draft; writing – review and editing. **Ekuwa Baisiwa Wilson:** Writing – review and editing. **Samuel Annang Darko:** Writing – review and editing. **Emmanuel Tamakloe:** Writing – review and editing.

## FUNDING INFORMATION

Centre for Plant Medicine Research, Mampong‐Akuapem.

## CONFLICT OF INTEREST STATEMENT

The authors declare no conflict of interest.

## CONSENT

Written informed consent was obtained from the patient to publish this report in accordance with the journal's patient consent policy.

## Data Availability

The data that supports the findings of this study is available from the corresponding author upon reasonable request.

## References

[1] Kumar A , Dowling WE , Román RG , et al. Status report on COVID‐19 vaccines development. Curr Infect Dis Rep. 2021;23(9):1‐12. 10.1007/s11908-021-00752-3 PMC8043838 33867863

[2] Margherita M , Giulia DA , Caterina S , Nicola C , Vanvitelli C‐G . Clinical presentation of COVID‐19: case series and review of literature. Int J Environ Res Public Health. 2020;17:5062. 32674450 10.3390/ijerph17145062 PMC7399865

[3] Provisional Standard Treatment Guidelines for Novel Coronavirus Infection . COVID‐19 Guidelines for Ghana. Ministry of Health; 2020.

[4] Yuki K , Fujiogi M , Koutsogiannaki S . COVID‐19 pathophysiology: a review. Clin Immunol. 2020;215:108427. 32325252 10.1016/j.clim.2020.108427 PMC7169933

[5] COVID‐19 Treatment Guidelines Panel . Coronavirus Disease 2019 (COVID‐19) Treatment Guidelines. National Institutes of Health. Accessed September 27, 2023. https://www.covid19treatmentguidelines.nih.gov/ 34003615

[6] Lone SA , Ahmad A . COVID‐19 pandemic – an African perspective. Emerg Microbes Infect. 2020;9(1):1300‐1308. 32458760 10.1080/22221751.2020.1775132 PMC7473237

[7] WHO , 2020. Accessed October 15, 2021. https://www.afro.who.int/news/who‐supports‐scientifically‐proven‐traditional‐medicine?gclid=Cj0KCQiAjJOQBhCkARIsAEKMtO3uvF9rJZjebvPwe666Gy_MvA80kny2Pg5yGyWmmyPog5nozCDfPV0aApYuEALw_wcB.

[8] Subaprya R , Nagini S . Medicinal properties of neem leaves: a review. Curr Med Chem Anticancer Agents. 2005;5(2):149‐156. 15777222 10.2174/1568011053174828

[9] Mohammed AA . Therapeutics role of *Azadirachta indica* (neem) and their active constituents in diseases prevention and treatment. Evid Based Complement Alternat Med. 2016;2016:7382506. 27034694 10.1155/2016/7382506 PMC4791507

[10] Domfeh SA , Narkwa PW , Quaye O , et al. Cryptolepine and Nibima inhibit hepatitis B virus replication. Sci Afr. 2021;13:e00942.

[11] Attah AF , Fagbemi AA , Olubiyi O , et al. Therapeutic potentials of antiviral plants used in traditional African medicine with COVID‐19 in focus: a Nigerian perspective. Front Pharmacol. 2021;12:596855. doi:10.3389/fphar.2021.596855 33981214 PMC8108136

[12] CDC , 2019. Accessed October 17, 2021. https://www.cdc.gov/coronavirus/2019‐ncov/variants/index.html

[13] COVID‐19 Treatment Guidelines Panel . Coronavirus Disease 2019(COVID‐19) Treatment Guidelines. National Institutes of Health. Accessed: October 15, 2021. https://www.covid19treatmentguidelines.nih.gov/ 34003615

[14] Borkotoky S , Banerjee M . A computational prediction of SARS‐CoV‐2 structural protein inhibitors from *Azadirachta indica* (Neem). J Biomol Struct Dyn. 2021;39(11):4111‐4121. doi:10.1080/07391102.2020.1774419 32462988 PMC7311162

[15] Borquaye LS , Gasu EN , Ampomah GB , et al. Alkaloids from *Cryptolepis sanguinolentaas* potential inhibitors of SARS‐CoV‐2 viral proteins: An in silico study. Biomed Res Int. 2020;2020:5324560. 33029513 10.1155/2020/5324560 PMC7512045

[16] Biswas D , Nandy S , Mukherjee A , Pandey DK , Dey A . *Moringa oleifera* Lam. and derived phytochemicals as promising antiviral agents: a review. S Afr J Bot. 2020;1(129):272‐282.

